# Histopathological Ratios to Predict Gleason Score Agreement between Biopsy and Radical Prostatectomy

**DOI:** 10.3390/diagnostics11010010

**Published:** 2020-12-23

**Authors:** Vincenzo Fiorentino, Maurizio Martini, Marco Dell’Aquila, Teresa Musarra, Ersilia Orticelli, Luigi Maria Larocca, Ernesto Rossi, Angelo Totaro, Francesco Pinto, Niccolò Lenci, Valerio Di Paola, Riccardo Manfredi, Pier Francesco Bassi, Francesco Pierconti

**Affiliations:** 1 Institute of Pathology, Università Cattolica del S. Cuore, Fondazione Policlinico “A. Gemelli”, 00168 Rome, Italy; vincenzof.89@hotmail.it (V.F.); maurizio.martini@unicatt.it (M.M.); mzrk07@gmail.com (M.D.); teresamusarra88@gmail.com (T.M.); orticelliersilia@gmail.com (E.O.); luigimaria.larocca@unicatt.it (L.M.L.); 2 Institute of Medical Oncology, Università Cattolica del S. Cuore, Fondazione Policlinico “A. Gemelli”, 00168 Rome, Italy; ernestorossi.rm@gmail.com; 3 Institute of Urology, Università Cattolica del S. Cuore, Fondazione Policlinico “A. Gemelli”, 00168 Rome, Italy; angelo.totaro@policlinicogemelli.it (A.T.); francesco.pinto@policlinicogemelli.it (F.P.); lenci.niccolo@live.com (N.L.); bassipf@gmail.com (P.F.B.); 4 Department of Radiology, Università Cattolica del S. Cuore, Fondazione Policlinico “A. Gemelli”, 00168 Rome, Italy; valerio.dipaola@policlinicogemelli.it (V.D.P.); riccardo.manfredi@policlinicogemelli.it (R.M.)

**Keywords:** active surveillance, Gleason score, needle biopsy, prostate cancer, radical prostatectomy

## Abstract

Biopsy proven Gleason score is essential to decide treatment modalities for prostate cancer, either surgical (radical prostatectomy) or non-surgical (active surveillance, watchful waiting, radiation therapy and hormone therapy). Several studies indicated that biopsy proven Gleason score may underestimate Gleason score at radical prostatectomy, hence we aimed to calculate the minimum length of biopsy cores needed to have Gleason score agreement. We evaluated 115 prostate cancer patients who underwent multiparametric magnetic resonance/transperineal ultrasonography fusion biopsy and subsequently, radical prostatectomy. Biopsy proven Gleason score was consistent with Gleason score at subsequent radical prostatectomy in 82.6% of patients, while in 17.4% of patients, Gleason score was higher at radical prostatectomy. Gleason score agreement showed a strong direct association with a ratio > 0.05 between the total volume of biopsies performed in tumor area and the volume of the corresponding tumor at radical prostatectomy. A significant association was also found with a ratio ≥ 0.0034 between the tumor volume in the biopsy and the volume of the corresponding tumor at radical prostatectomy and with a ratio ≥ 0.086 between the tumor volume in the biopsy and the total volume of biopsies performed in the tumor area. These results could be exploited to calculate the minimum length of biopsy cores needed to have a correct Gleason score estimation and therefore be used in fusion targeted biopsies with volume adjustments.

## 1. Introduction

Prostate cancer (PCa) is the second most frequent malignancy (after lung cancer) in men and the fifth leading cause of death worldwide [1]. In its initial phase, the tumor is asymptomatic and could only be detected by digital rectal examination (DRE), abnormal increase in prostate-specific antigen (PSA) plasmatic levels or by transrectal ultrasonography (TRUS). If any alteration is found, a TRUS-guided biopsy (TRUS-bx) must be performed. Histological examination of bioptic specimen provides tumor data of the Gleason score (GS), a grading system based on glandular architecture of prostate adenocarcinoma that defines five histological grades with decreasing differentiation. GS is associated with tumor prognosis and biopsy proven Gleason score (bx-GS) is an important tool to decide treatment modalities, either surgical (radical prostatectomy (RP)) or non-surgical (active surveillance (AS), watchful waiting, radiation therapy and hormone therapy) [2,3].

In 2014, the ISUP (International Society of Urological Pathology) and the WHO (World Health Organization) adopted a new grading system composed of five prognostic grade groups, as follows: Gleason score ≤ 6 (prognostic grade group 1), Gleason score 3 + 4 = 7 (prognostic grade group 2), Gleason score 4 + 3 = 7 (prognostic grade group 3), Gleason score 4 + 4 = 8 (prognostic grade group 4) and Gleason score 9–10 (prognostic grade group 5) [4]. This simple grading system was originally proposed in 2013 by Pierorazio et al. [5] and proved to accurately stratify patients to predict clinical outcomes. It was then validated on a larger cohort from five institutions from the Unites States and Europe, demonstrating a distinct biochemical recurrence-free survival between the grade groups [6].

In the last few years, there has been an increasing use of multiparametric magnetic resonance imaging (mpMRI) of prostate to localize suspicious areas, which could be targeted-biopsied under real-time ultrasound guidance. mpMRI/TRUS fusion targeted biopsies have proven optimal detection of significant prostate cancer while reducing diagnosis of insignificant PCa [7]. Previous studies have shown no common agreement on the meaning of the term “clinically significant” except for the fact that it has a GS ≥ 7, has a more aggressive clinical behavior and is not suitable for AS. This definition is also used to identify significant lesions with the Prostate Imaging Reporting and Data System (PIRADS), a scoring system created in 2012 to unify the interpretation and reporting of prostate MRI findings [8], and recently revised in 2019 (PI-RADSv2.1) to allow an efficient and reproducible detection of clinically significant cancer with mpMRI [9].

This system is based on a scale from 1 to 5 with increasing suspicion of clinically significant cancer: very low in PIRADS 1 category, low in PIRADS 2 category, intermediate in PIRADS 3 category, high in PIRADS 4 category and very high in PIRADS 5 category. Each category is assigned according to specific imaging features in each imaging sequence, including T2-weighted imaging, diffusion weighted imaging (DWI) and dynamic contrast-enhanced imaging (DCE).

Several studies have shown the utility of PIRADSv2 in prostate cancer detection and evaluation and there is growing evidence showing a low incidence of clinically significant disease in PIRADS 3 nodules (intermediate assessment category) [10,11,12]. Nevertheless, correlating bx-GSs with GSs obtained from RPs indicated a bx-GS upgrading rate between 30% and 50% for systematic biopsies [10,13] and between 7% and 22% for the combination of fusion biopsy and systematic biopsy [14]. Notably, GS upgrading (GSU) after RP is highly associated with a risk of extracapsular extension, biochemical recurrence and cancer-specific mortality [3].

For this reason, in the last few years, different research groups evaluated the variables that could predict a change in GS from biopsy to RP specimen. The main reasons for GS discrepancies comprise pathologist grading errors, borderline grades, sampling errors [15] and the multifocal disposition of PCa: different areas within prostatic tissue could have different grades [16].

To overcome these issues, in this study, we hypothesized that Gleason score agreement could depend on the relationship between tissue biopsy parameters (such as the total volume of the biopsies in the tumor area and the tumor volume in the biopsies) and the volume of the same tumor at RP.

We therefore calculated the ratios between the aforementioned parameters and found a correlation between these ratios and a correct GS estimation. All patients underwent mpMRI/TRUS fusion targeted biopsies, and for each area, we compared bx-GS with GS in the corresponding area at RP and then correlated the agreement of GS with three ratios: the ratio between the total volume of biopsies performed in tumor area (BTA) and the volume of the same tumor area identified at RP (TV), the ratio between the overall tumor volume in the biopsies (BTV) and TV and the ratio between BTV and BTA. We then established a cut-off value for each ratio above which the concordance of the GS was more probable. Finally, based on the aforementioned cut-off values, we devised formulas to calculate the minimum length of biopsy cores necessary to reach Gleason score agreement.

## 2. Materials and Methods

The present study represents a retrospective analysis of 115 patients with high clinical suspicion for PCa (i.e., elevated or rising PSA and/or suspicious DRE) who underwent mpMRI/TRUS fusion biopsy after a positive pre-biopsy mpMRI scan between November 2017 and February 2020 at our institution (Fondazione Policlinico Universitario Agostino Gemelli, Rome, Italy). All the analyzed data were collected as part of the routine diagnosis and treatment procedures at our institution.

Given the low incidence of clinically significant tumors in PIRADS 3 lesions, as previously discussed, only PIRADSv2.1 score ≥ 4 lesions were considered as positive scans and therefore targeted-biopsied. All patients were biopsy-naïve: none of them had undergone previous prostate mappings.

MpMRI in this study was performed using a 1.5 Tesla Achieva (Philips Healthcare, Best, Netherlands) and an integrated endorectal-pelvic phased-array coil (MR Innerva, Medrad, Pittsburgh, PA, USA). Multiplanar T2-weighted images (T2WI), diffusion-weighted imaging (DWI) with two b values (800 and 1400 s/mm^2^), dynamic gadolinium contrast-enhanced imaging (DCE) and delayed T1-weighted sequences with fat suppression were acquired, according to the Imaging protocol suggested by the PIRADSv2.1 [9,10,17]. The slice thickness of the T2WI of mpMRI was 3.5 mm.

MpMRI images were analyzed by two experienced radiologists and reported according to PIRADSv2.1 [9]. As already discussed, imaged lesions with a PIRADSv2.1 score ≥ 4 were targeted, and 4 to 6 specimens were obtained from each lesion. In case of multifocal disease, only the index lesion (IL) was targeted: IL was defined as the lesion with the highest PIRADSv2.1 score or the largest lesion in the presence of more than one lesion with the same score [14].

In case of multifocal disease, only the index lesion was targeted because it was considered as the most suspicious, and this statement is supported by literature data showing that, in case of multifocality, MRI is often able to detect the worst focus of cancer [18]. Moreover, increasing evidence shows that in multifocal disease, the dominant (or index) tumor may drive biologic behavior and that it may be the source of progenitor cells in later metastases [19,20].

Moreover, each patient concomitantly underwent a standard transperineal 12-core random systematic biopsy (avoiding IL) in accordance with EAU-ESTRO-ESUR-SIOG (European Association of Urology—European Society for Radiotherapy and Oncology—European Society of Urogenital Radiology—International Society of Geriatric Oncology) guidelines [21]. In this procedure, the horizontal section of the prostate was divided into 12 areas symmetrically distributed at the base, mid-gland and apex: the biopsy points were localized at the right and left apex, right and left mid-prostate, right and left base, right and left transition zone, right mid-lateral (2 cores) and left mid-lateral (2 cores) [22].

A software registration fusion approach was used to biopsy mpMRI visualized lesions: all targets were marked and superimposed with TRUS image before sampling [23]. Imaging fusion was obtained through a multimodality fusion imaging system (Virtual Navigator System and MyLab^™^Twice, Esaote SpA, Genoa, Italy). Two experienced urologists (A.T. and F.Po.) performed fusion biopsies and had access to all mpMRI data with radiologist-marked detected lesions. All targets were transperineally sampled under live TRUS visualization and biopsy cores were obtained using a standard 18-gauge needle and a biopsy gun. The core diameter of the needle was 1.02 mm [7,24].

All patients underwent robot-assisted laparoscopic prostatectomy (RALP) within 3 months from the biopsy, preventing potential grade progression between procedures. Extended pelvic lymph node dissection was performed based on their class of risk according to EAU-ESTRO-ESUR-SIOG guidelines [21].

Exclusion criteria included patients with a history of neoadjuvant hormonal therapy or chemoradiotherapy before RP surgery, active surveillance, previous surgery for benign prostatic hyperplasia (BPH), a core length less than 12 mm [25] and those patients who had incomplete medical records. Clinical and pathological data were recorded for all patients and bx-GSs were compared with their surgical counterparts. The grading of both biopsies and RPs was done by two expert genitourinary pathologists (F.Pi. and M.M.) and in doubtful cases, a third uro-pathologist (L.M.L.) was consulted to reach group consensus. The pathological classification followed the ISUP–WHO 2014 classification [4,26].

Upgrading and downgrading were respectively defined as an increase and a decrease from one prognostic GS grade group to another in pathological specimens after RP [2]. Pathological staging was performed according to the eighth edition of TNM (Tumor, Node, Metastasis) Classification of Malignant Tumors [27]. BTA was calculated by multiplying the area of needle section (i.e., 0.0082 cm^2^) by the length of obtained specimen, and BTV was calculated by multiplying the aforementioned area by tumor length present in the specimen.

RP specimens were examined and reviewed based on the protocol described by Montironi et al., in 2002, using whole mount sections, and tumor volume (TV) was determined by the grid method [28]. For each lesion identified by MpMRI, we calculated BTA, BTV and TV, then we calculated the ratios between these volumes and identified the best cut-off scores to have GS agreement.

The selection of cut-off scores was based on ROC analysis (Receiver Operating Characteristics (ROC) curve analysis). At each score, the sensitivity and specificity values were plotted, thus generating a ROC curve. The score located closest to the point with both maximum sensitivity and specificity on the curve (0.0, 1.0) was selected as the cut-off score, leading to the greatest number of tumors which were correctly classified as having or not having the outcome. Area under the ROC curves summarizes the discriminatory power of each ratio for the outcome, with values of 0.5 indicating low power and those closer to 1.0 indicating higher power (see Supplementary Table S1).

To verify whether the distribution of our data was normal or not, we performed a Shapiro–Wilk test of normality and we found that the population was not normally distributed (data not shown). Given the non-normal distribution, we opted for the use of a non-parametric test and we felt that Fisher’s exact test was the most appropriate to assess associations between categorical variables (i.e., to establish the validity of our cut-off values). This test in fact allowed us to create a contingency table and assess the correlation between our cut-off values and a correct Gleason score estimation. Consequently, we were able to establish a cut-off value for each ratio above which the concordance of the Gleason score was more probable.

Quantitative outcomes were apprised through descriptive statistics (mean ± standard deviation (SD)). Statistical analysis was performed using MedCalc version 10.2.0.0 (StataCorp LP, College Station, TX, USA) and the GraphPad-Prism 5 software (Graph Pad Software, San Diego, CA, USA). The statistically significant level was considered *p* < 0.05.

## 3. Results

The average patient age was 65.1 ± 7.15 years. The mean PSA of the patients before biopsy was 12.5 ± 4.9 ng/dL. The mean nodule volume was 0.865 ± 0.99 cm^3^. Multifocal disease was present in 79 out of 115 patients (68.7%) and the mean number of nodules at RP was 2.2 ± 1. Pathological stage and other pathological data were recorded as shown in Table 1.

All prostate specimens of the patients included in the study were reported as prostate acinar adenocarcinoma. In 95 (82.6%) patients, bx-GS was consistent with RP-GS, while in 20 (17.4%) patients, RP-GS was higher than bx-GS. None of the patients resulted in a RP-GS lower than bx-GS. Biopsy pathology was ISUP 1 in 85 patients (74%), ISUP 2 in 15 patients (13%), ISUP 3 in 10 patients (9%) and ISUP 4 in 5 patients (4%). RP pathology was ISUP 1 in 70 patients (61%), ISUP 2 in 15 patients (13%), ISUP 3 in 10 patients (9%) and ISUP 4 in 20 patients (17%). After RP pathology, 10 patients were upgraded from ISUP 1 to ISUP 4, while 5 from ISUP 1 to ISUP 2 and 5 from ISUP 2 to ISUP 4, respectively. Bioptic and RP material ISUP classifications are shown in Table 1 [26].

**Figure 1:**
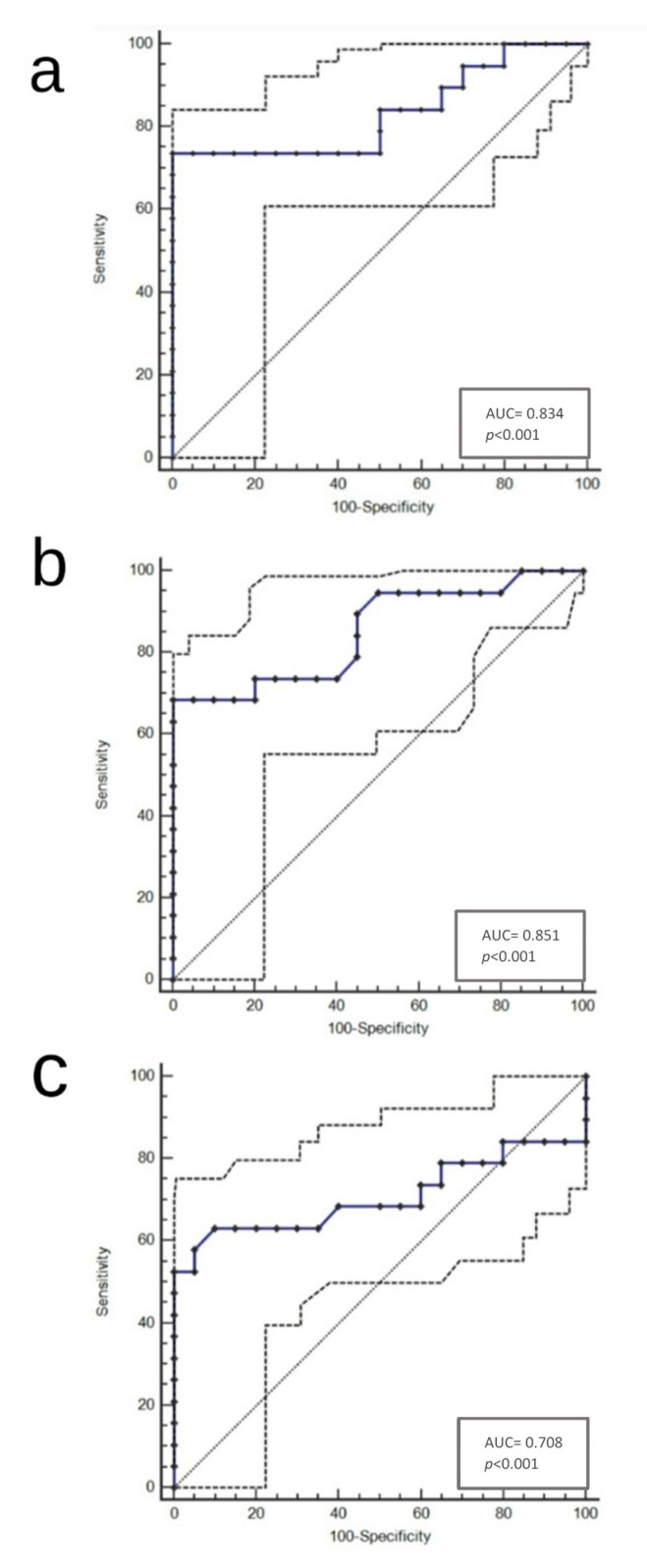
(**a**–**c**): ROC curves for BTA/TV (cut-off > 0.05) (**a**), BTV/TV (cut-off ≥ 0.0034) (**b**) and BTV/BTA (cut-off ≥ 0.086) (**c**). Abbreviations: ROC, Receiver Operating Characteristic.

Our results suggested that a BTA/TV ratio > 0.05 represents the best cut-off score in the prediction of a correct GS (*p* < 0.001; AUC = 0.834; 95% CI (Confidence Interval) from 0.753 to 0.897; Youden index J = 0.7368; Sensitivity 73.68%; Specificity 100%; Figure 1a and Supplementary Table S1), while a BTV/TV ratio ≥ 0.0034 has been identified as the best cut-off score in the prediction of a correct GS (*p* < 0.0001; AUC = 0.851; 95% CI from 0.773 to 0.911; Youden index J = 0.6842; Sensitivity 68,42%; Specificity 100%; Figure 1b and Supplementary Table S1). Moreover, we found that a BTV/BTA ratio ≥ 0.086 was the best cut-off score in the prediction of a correct GS (*p* < 0.0001; AUC = 0.708; 95% CI from 0.616 to 0.789; Youden index J = 0.5316; Sensitivity 63.16%; Specificity 90%; Figure 1c and Supplementary Table S1).

Using Fisher’s exact test, we found that a correct GS evaluation had a strong direct association with a BTA/TV ratio > 0.05 (*p* = 0.0015), with a BTV/TV ratio ≥ 0.0034 (*p* = 0.0002) and with a BTV/BTA ratio ≥ 0.086 (*p* = 0.0115).

The aforementioned correlations are shown in Table 2 and formulas to calculate the minimum length of biopsy specimens are shown in the Equations.

(1) $$0.05=\frac{k*\text{LTA}}{\text{TV}}$$

(2) $$0.0034=\frac{k*\text{TLB}}{\text{TV}}$$

(3) $$0.086=\frac{\text{TLB}}{\text{LTA}}$$

Formulas to calculate the minimum length of biopsy specimens for each ratio. Abbreviations: k = 0.0082 cm^2^ (area of needle section); LTA, length of biopsies performed in tumor area (cm); TLB, overall tumor length in the biopsies (cm); TV, tumor volume at RP (cm^3^).

## 4. Discussion

PCa treatment modalities are mainly based on pathologic information obtained from needle biopsies, that should coincide with those obtained from RPs. On this basis, GS preoperative evaluation of PCa patients has a pivotal importance in therapeutic conduct and essential prognostic implications, being the most effective prognostic method to predict patients’ clinical outcomes. GS is crucial to determine an appropriate therapeutic strategy for patients with PCa and its evaluation should be as accurate as possible. GSU after RP is a common finding in clinical practice and many efforts have been made to increase the reliability of GS determination at needle biopsy. Divrik et al. [29] and Yang et al. [30] showed that increasing the number of biopsies, beyond its superior diagnostic accuracy, strengthens GS accuracy for predicting the final PCa grade. Divrik et al. also hypothesized that the evaluation of specimens by an expert may have an additional positive effect on concordance. Moreover, further studies have investigated possible predictors of GSU, like a small prostate volume [31], pre-biopsy PSA and highest percentage of cancer in the biopsy [32].

In our analysis, the percentage of GSU was 17.4%, consistent with literature data showing that the combination of fusion biopsy and systematic biopsy has an upgrading rate between 7% and 22% [14]. Our present results are also in accordance with literature for GS downgrading in fusion targeted biopsies, showing that in no patient was RP-GS lower than bx-GS, as previously reported by Porpiglia et al. [33], who showed a 1% downgrading rate between fusion targeted biopsies and RP. Moreover, the percentage of multifocal disease was 68.7%, consistent with literature data showing multiple PCa foci in approximately 70% of patients, even with low-risk disease [34].

Nevertheless, this study represents the first effort to investigate the relationship between the volumes of bioptic specimens obtained from PIRADSv2.1 score ≥ 4 areas and tumor volumes of the same areas identified at RP, in order to calculate the minimum length of biopsy cores necessary to have a correct GS estimation. We therefore established cut-off values above which GS concordance was more probable, thus providing urologists with an effective tool to perform an adequate bioptic sampling of prostatic nodules. As demonstrated by Baco et al., mpMRI/TRUS fusion biopsy can effectively identify the position of clinically relevant PCa with a 95% concordance between tumor location on biopsy and RP [35]. Given the high diagnostic accuracy and excellent sensitivity of mpMRI with real-time ultrasound, our ratios, based on pathological data, may be used in fusion targeted biopsies to calculate the minimum length of biopsy cores necessary to avoid GSU. The only limit to overcome would be tumor volume estimation; in fact, as shown by Baco et al., there is a 5.9% tumor volume mean underestimation at mpMRI, and this finding was confirmed by Radtke et al. [7], who found a 36% mean volume underestimation. To overcome this limit, Radtke et al. used a shrinkage factor (1.15) to improve mpMRI tumor volume estimations, with a resulting decreased (20%) mean volume underestimation. Therefore, by using a simple formula, as shown in the Equations, clinicians could calculate the minimum length of bioptic specimens enough to have GS agreement. This is especially true for the formula in Equation (1), because it exploits variables that can be evaluated by the clinician at the time of biopsy sampling (i.e., the length of biopsies performed in tumor area (LTA) and the tumor volume (TV) estimated at mpMRI using the aforementioned shrinkage factor). On the other hand, the Equations (2) and (3), considering the overall tumor length in the biopsies (TLB), can be used only after microscopic evaluation of biopsy specimens. Nevertheless, our work could have immediate practical implications, resulting in less pathologic upgrading with an improved risk stratification of patients with prostatic adenocarcinoma and a better therapeutic conduct.

Our only purpose was to investigate the relationship between tissue biopsy parameters (such as the total volume of the biopsies in the tumor area and the tumor volume in the biopsies) and whole gland specimen pathological findings (i.e., tumor volume at radical prostatectomy). The comparison of pathological findings between biopsy and whole-gland specimen was evaluated exclusively in target biopsies and systematic biopsy was mentioned only because it was a part of the routine diagnostic procedures at our institution. We considered only the bioptic specimens obtained from PIRADSv2.1 score ≥ 4 areas because we wanted to increase the likelihood of extensively sampling suspicious areas, thus increasing the probability of providing a correct Gleason score. Therefore, in our view, there was no need to compare pathological findings between biopsy and whole-gland specimen separately in target and systematic biopsy since Gleason score agreement exclusively depends on the quantity of samples taken in neoplastic areas and not on the type of biopsy approach adopted (i.e., even random biopsies can estimate a correct Gleason score if a nodule sampling meets our parameters).

Finally, since the biopsy core length determines the quality of a specimen and given the impact of core length on cancer detection [25], we considered only the biopsy samples with a suitable length (i.e., 12 mm) and observed that our ratios worked regardless of the length of biopsy specimen, being the only predictors of Gleason score agreement.

Notwithstanding the relatively small sample size, single-center evaluation with the lack of a validation series and the non-flexible number of the systematic biopsy (although the prostate volume ranged from 11.5 to 137.4 cc, the number of samples in systematic biopsies was always the same), our study paved the way for a new method to evaluate biopsy cores to yield correct GS estimation. Considering that GS is one of the key parameters to inform a decision of active surveillance vs. treatment, increasing the accuracy of GS assessment is of potential clinical impact. We therefore attempted to provide useful practical information for performance of mpMRI/TRUS fusion biopsies, minimizing GS upgrading.

Large, prospective cohort studies with long-term follow-up will be needed to corroborate our findings.

## 5. Conclusions

In this study, we investigated the relationship between the volumes of bioptic specimens obtained from PIRADSv2.1 score ≥ 4 areas and tumor volumes of the same areas identified at RP, in order to calculate the minimum length of biopsy cores necessary to have a correct GS estimation. This is the first attempt to investigate the relationship between the volumes of bioptic specimens and corresponding tumor volumes at RP, in order to devise a formula useful for a correct GS estimation.

Our results represent a starting point for further studies with a larger cohort of patients to strengthen and provide a wider application to our conclusions.

## Figures and Tables

**Table 1:** Clinical and pathological patients’ characteristics.

Number of Patients	115 (100%)
Mean age in years ± SD (range)	65.1 ± 7.15 (49–77)
Mean PSA at biopsy (ng/mL) ± SD (range)	12.5 ± 4.9 (6.5–27)
Multifocal disease	79/115 (68.7%)
Mean number of nodules ± SD (range)	2.2 ± 1 (1–4)
Mean prostate weight (g) ± SD (range)	53.5 ± 25.75 (26.1–155)
Mean prostate volume (cm^3^) ± SD (range)	43.56 ± 29.4 (11.5–137.4)
Mean TV (cm^3^) ± SD (range)	0.865 ± 0.99 (0.016–3.545)
Mean BTA (cm^3^) ± SD (range)	0.238 ± 0.88 (0.00574–4.345)
Mean BTV (cm^3^) ± SD (range)	0.00698 ± 0.0076 (0.000416–0.03)
Pathological stage, n (%)	pT2a/b: 20 (17–4)
	pT2c: 70 (60.9)
	pT3a: 20 (17.4)
	pT3b: 5 (4.3)
Lymph node status, n (%)	pN0: 50 (43.5)
	pN1: 0 (0)
	pNx: 65 (56.5)
Biopsy Gleason score, n (%)	ISUP 1 (3 + 3): 85 (74)
	ISUP 2 (3 + 4): 15 (13)
	ISUP 3 (4 + 3): 10 (9)
	ISUP 4 (4 + 4, 3 + 5, 5 + 3): 5 (4)
Radical prostatectomy Gleason score, n (%)	ISUP 1 (3 + 3): 70 (61)
	ISUP 2 (3 + 4): 15 (13)
	ISUP 3 (4 + 3): 10 (9)
	ISUP 4 (4 + 4, 3 + 5, 5 + 3): 20 (17)

Abbreviations: PSA, Prostate Specific Antigen; ISUP, International Society of Urological Pathology; SD, standard deviation; BTA, volume of biopsies performed in tumor area; BTV, overall tumor volume in the biopsies; TV, tumor volume at RP.

**Table 2:** Correlation among ratios between bioptic and surgical tumor volumes and GS (Gleason Score) agreement.

Ratios	Cut-Off	Upgrade (*n* = 20)	No Upgrade (*n* = 95)	*p*-Value ^a^
BTA/TV	>0.05 ≤0.05	7 13	70 25	*p* = 0.0015
BTV/TV	≥0.0034 <0.0034	12 8	90 5	*p* = 0.0002

^a^ Fisher’s exact test *p*-value. Abbreviations: BTA, volume of biopsies performed in tumor area; BTV, overall tumor volume in the biopsies; TV, tumor volume at RP (radical prostatectomy).

## Data Availability

The data presented in this study are available on request from the corresponding author.

## Supplementary Materials

The following are available online at https://www.mdpi.com/2075-4418/11/1/10/s1, Table S1: Performance parameters of ROC curve analysis.
