# Supplementary material for: Histopathological Ratios to Predict Gleason Score Agreement between Biopsy and Radical Prostatectomy
Source: Diagnostics (Basel). 2020 Dec 23;11(1):10. doi: 10.3390/diagnostics11010010 (PMC7822416; doi:10.3390/diagnostics11010010)
Supplement: Supplementary file 1 [file diagnostics-11-00010-s001.pdf]

**Table S1.** Performance parameters of ROC curve analysis.

| | |
|---|---|
| **Sample size** | 115 |
| **GS concordant group** | 95 (82.6%) |
| **GS discordant group** | 20 (17.4%) |
| **Disease prevalence (%)** | unknown |
| **BTA/TV (cut-off > 0.05)** | |
| AUC | 0.834 |
| Standard Error | 0.0384 |
| 95% Confidence interval | 0.753 to 0.897 |
| z statistic | 8.708 |
| Significance level P (Area = 0.5) | <0.0001 |
| Youden index J | 0.7368 |
| Associated criterion | >0.07 |
| Sensitivity (%) | 73.68 |
| Specificity (%) | 100 |
| **BTV/TV (cut-off ≥ 0.0034)** | |
| AUC | 0.851 |
| Standard Error | 0.0380 |
| 95% Confidence interval | 0.773 to 0.911 |
| z statistic | 9.235 |
| Significance level P (Area = 0.5) | <0.0001 |
| Youden index J | 0.6842 |
| Associated criterion | >0.0085 |
| Sensitivity (%) | 68.42 |
| Specificity (%) | 100 |
| **BTV/TV (cut-off ≥ 0.086)** | |
| AUC | 0.708 |
| Standard Error | 0.0458 |
| 95% Confidence interval | 0.616 to 0.789 |
| z statistic | 4.537 |
| Significance level P (Area = 0.5) | <0.0001 |
| Youden index J | 0.5316 |
| Associated criterion | >0.095 |
| Sensitivity (%) | 63.16 |
| Specificity (%) | 90.00 |

Abbreviations: ROC, Receiver Operating Characteristics; AUC, Area under the ROC curve; GS, Gleason Score; BTA, volume of biopsies performed in tumor area; BTV, overall tumor volume in the biopsies; TV, tumor volume at RP.
